# T_1_ mapping in cardiac MRI

**DOI:** 10.1007/s10741-017-9627-2

**Published:** 2017-06-16

**Authors:** Dina Radenkovic, Sebastian Weingärtner, Lewis Ricketts, James C. Moon, Gabriella Captur

**Affiliations:** 1 Barts Heart Center, The Cardiovascular Magnetic Resonance Imaging Unit, St Bartholomew’s Hospital, West Smithfield, London, UK; 2 University College London Medical School, Bloomsbury Campus, Gower Street, London, UK; 3 Computer Assisted Clinical Medicine, University Medical Center Mannheim, Medical Faculty Mannheim, Heidelberg University, Theodor-Kutzer-Ufer, Mannheim, Germany; 4 Department of Medicine Cardiology, University Medical Center Mannheim, Medical Faculty Mannheim, Heidelberg University, Mannheim, Germany; 5 Department of Electrical and Computer Engineering, University of Minnesota, Minneapolis, MN USA; 6 NIHR University College London Hospitals Biomedical Research Center, Tottenham Court Road, London, UK; 7 UCL Institute of Cardiovascular Science, University College London, London, UK

**Keywords:** T_1_ mapping, Extracellular volume, Myocardial disease

## Abstract

Quantitative myocardial and blood T_1_ have recently achieved clinical utility in numerous pathologies, as they provide non-invasive tissue characterization with the potential to replace invasive biopsy. Native T_1_ time (no contrast agent), changes with myocardial extracellular water (edema, focal or diffuse fibrosis), fat, iron, and amyloid protein content. After contrast, the extracellular volume fraction (ECV) estimates the size of the extracellular space and identifies interstitial disease. Spatially resolved quantification of these biomarkers (so-called T_1_ mapping and ECV mapping) are steadily becoming diagnostic and prognostically useful tests for several heart muscle diseases, influencing clinical decision-making with a pending second consensus statement due mid-2017. This review outlines the physics involved in estimating T_1_ times and summarizes the disease-specific clinical and research impacts of T_1_ and ECV to date. We conclude by highlighting some of the remaining challenges such as their community-wide delivery, quality control, and standardization for clinical practice.

## Introduction

In magnetic resonance imaging, the longitudinal (spin-lattice) relaxation time (T_1_) is a fundamental tissue property, now measurable in the myocardium using cardiac T_1_ mapping sequences. Cardiovascular magnetic resonance (CMR) research data accrued in both animals and humans convincingly demonstrate that native T_1_, in the absence of gadolinium-based contrast agents (GBCA), lengthens with interstitial expansion caused by edema, infarction, amyloid infiltration, and fibrosis [1]. Conversely, native T_1_ shortens in the presence of fat and iron accumulation. The left ventricular (LV) myocardial native T_1_ signal, from a single region of interest on a T_1_ map, could therefore serve as a simple, on-the-fly, non-invasive discriminator of heart muscle health and disease.

**Fig. 1:**
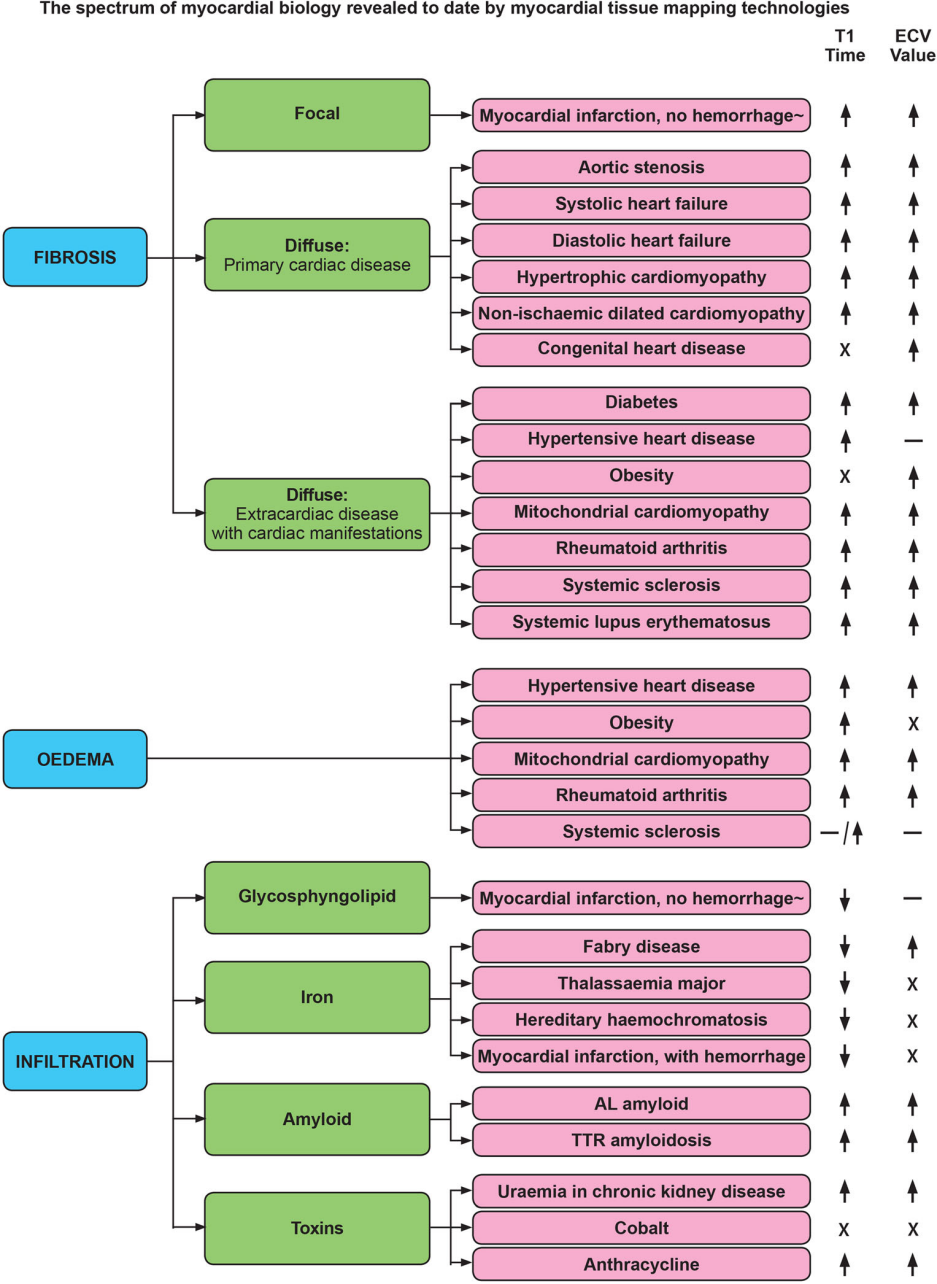
Summary of myocardial biological changes inferred by T_1_ mapping technologies. ↑ = significant increase; ↓ = significant decrease; − = no significant change; X = no data available. *ECV* extracellular volume, *AL Amyloid* amyloid light chain, *TTR amyloidosis* transthyretin amyloidosis

T_1_-weighted signal also forms the basis of the late gadolinium enhancement (LGE) imaging technique. This technique was the most disruptive tissue characterization method. LGE can quantify focal scar and fibrosis in both ischemic and non-ischemic cardiomyopathies. It works by the principle that scarred tissue passively accumulates more GBCA which shortens its T_1_ compared to adjacent normal healthy myocardium, and this is visible with a particular imaging sequence (inversion recovery) [2]. T_1_ mapping adds to this. It has evolved from T_1_-weighted imaging, to native T_1_ measurement alone, to post-GBCA T_1_ measurement in isolation, or through the partition coefficient to measurement of the extracellular volume (ECV) [3]. The latter is when T_1_ is measured before and after GBCA using a correction for the hematocrit (measured separately or in-line automated) [4, 5]. Native T_1_ and ECV permit earlier diagnosis and quantitative assessment of focal as well as diffuse myocardial disease (Fig. 1). T_1_ mapping by CMR describes the pixel-wise quantification of the spin-lattice relaxation time in order to provide a quantitative tissue characterization that is commonly viewed as a color-coded map of the heart. T_1_ maps are most commonly derived from a series of T_1_-weighted images, sampling the T_1_ recovery curve after one or more initial preparation pulses.

This review outlines the basic physics of T_1_ mapping and discusses disease-specific clinical and research impacts of T_1_ and ECV to date. We conclude by highlighting the challenges of community-wide delivery, quality control, and standardization in clinical practice.

## Essential physics and evolution of T_1_ mapping sequences

**Fig. 2:**
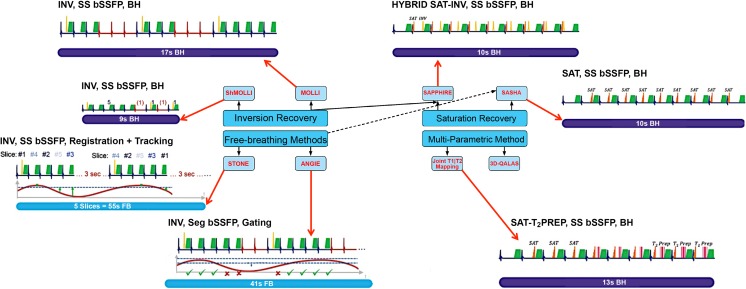
Illustrated overview of T_1_ mapping acquisition strategies. The techniques are divided into four major groups: MOLLI, saturation recovery, free-breathing methods, and multi-parameter imaging. The graphs diagrammatically represent the inversion pulse and acquisition times across heartbeats. Diaphragmatic movement during image acquisition is shown for the free-breathing methods STONE and ANGIE. Technical details of described T_1_ mapping acquisition strategies are described in Table 1. *ANGIE* Accelerated and Navigator-Gated Look-Locker Imaging for Cardiac T_1_ Estimation, *BH* breath-hold, *bSSFP* balanced steady-state free precession, *3D-QALAS* three-dimensional-QuAntification using an interleaved Look-Locker Acquisition Sequence with T2 preparation pulse, *INV* inversion, *FB* free-breathing, *MOLLI* Modified Look-Locker Inversion, *Prep* preparation, *SAPPHIRE* Saturation Pulse Prepared Heart-Rate Independent Inversion REcovery Sequence, *SASHA* saturation recovery single shot acquisition, *SAT* saturation, *Seg* segmented, *ShMOLLI* shortened MOLLI, *SS* single shot, *STONE* slice-interleaved T_1_ mapping sequence

Broadly, T_1_ mapping sequences have three parts: (1) the T_1_ magnetization preparation pulse, (2) a single image acquisition (readout) after a variable delay, and (3) variable repetitions of (1) and (2) to sample the longitudinal magnetization recovery curve after the magnetization preparation. Raw images are then reconstructed by post-processing into a single T_1_ map using a theoretical model of the expected signal intensity [3] and with the help of various refinements such as respiratory motion compensation (Table 1 and Fig. 2).

**Table 1:** Overview of salient T_1_ mapping sequences comparing building plans, strengths, and limitations^a^

Sequence	Building plan: 3 integral parts			Strength	Limitation	Reference
	T_1_ preparation	Imaging readout	Respiratory motion compensation			
Original MOLLI	IR pulse over multiple heartbeats	Single-shot end-diastolic bSSFP	Single breath-hold	– High-quality T_1_ maps – Good precision (noise resilience) – Widely available – High inter-center reproducibility	– T_1_ time dependence on T_2_, MT, and sequence parameters – HR dependence	[6]
Fixed-recovery MOLLI	IR pulse over multiple heartbeats	Single-shot end-diastolic bSSFP	Single 11-s breath-hold	– Little HR variability – Separate optimization allows precision for both native and post-GBCA regimes	– Requires different protocols for native and post-GBCA scans	[7]
ShMOLLI	IR pulse over multiple heartbeats	Single-shot end-diastolic bSSFP	Single short 9-s breath-hold	– Short breath-holds via short rest periods of 1 heartbeat – Incomplete magnetization recovery compensated for by conditional data fit – Unified sequence for pre-/post-GBCA scanning – Little HR variability	– Low number of fit images available for use especially in native mapping – Vulnerable to mistriggering as sampling sparse	[8]
FLASH-MOLLI	IR pulse over multiple heartbeats	Single-shot end-diastolic FLASH	Single 11-s breath-hold	– Avoids off-resonance artifacts (good for high-field strengths) – No T_2_ dependence – Tailored fitting compensates for disruption of relaxation by FLASH pulses improving accuracy compared to original MOLLI	– Decreased SNR compared to SSFP schemes – Elaborate post-processing limits availability	[9]
TRASSI	IR pulse over multiple heartbeats	Radial golden-angle FLASH	Single short 5-s breath-hold	– Inherent properties of the radial acquisition, short breath-hold, and HR-adaptable acquisition window provide high resilience to motion artifacts – Tailored fit improves accuracy	– Potential blurring due to view-sharing across heartbeats – Little baseline data – Elaborate post-processing limits availability	[10]
SASHA	SR preparation over multiple heartbeats	Single-shot bSSFP	Single 10-s breath-hold	– Excellent accuracy as invariant to T_2_, MT, and inversion efficiency – Alternative reconstruction scheme has been proposed to trade off accuracy against precision – High-contrast imaging scheme available for free-breathing applications	– Still low precision compared to MOLLI – Low SNR baseline images more prone to artifacts – Low blood-myocardial imaging contrast makes post-processing with image registration challenging	[11]
SMART_1_ MAP	A series of single-point SR experiments	Single-shot bSSFP	Single breath-hold (13 heartbeats)	– Intra-scan heart rate insensitivity by adapting recovery time to changing heart rates by measuring heartbeats in real time – Good accuracy compared to MOLLI	– Limited data on in vivo clinical applicability; has yet to be validated at scale and on other vendor platforms	[12, 13]
SAPPHIRE	Hybrid SR/IR over multiple heartbeats	Single-shot bSSFP	Single 10-s breath-hold	– Good accuracy compared to MOLLI – Improved precision compared to SASHA	– Lower precision compared to MOLLI – Low SNR images are prone to artifacts	[14]
STONE	IR pulse over multiple heartbeats	Single-shot bSSFP	Interleaved multi (5)-slice 55 s free-breathing + registration + real-time slice tracking	– No rest periods between breath-holds as free-breathing improves patient comfort – Improved accuracy due to slice-interleaved scanning	– Potential for slice-tracking failure in heavy breathing patients – Perturbation of blood T_1_ times due to crosstalk between slices	[15]
ANGIE	IR pulse over multiple heartbeats	Segmented bSSFP	41 s free-breathing + diaphragmatic navigator gating	– No rest periods between breath-holds as free-breathing improves patient comfort – Enables high-resolution scans – Motion compensation robust to heavy breathing	– Accuracy comparable to MOLLI (not superior) – Elaborate compressed sensing reconstruction needed before the fit which limits availability	[16]

*ANGIE* Accelerated and Navigator-Gated Look-Locker Imaging for cardiac T_1_ Estimation, *bSSFP* balanced steady-state free precession, *FLASH* fast low-angle shot, *GBCA* gadolinium-based contrast agents, *HR* heart rate, *IR* inversion recovery, *MOLLI* MOdified Look-Locker Inversion Recovery, *MT* magnetization transfer, *s* second(s), *SAPPHIRE* SAturation Pulse Prepared Heart-Rate Independent Inversion REcovery Sequence, *SASHA* SAturation Recovery Single SHot Acquisition, *ShMOLLI* shortened MOLLI, *SNR* signal-to-noise ratio, *SR* saturation recovery, *STONE* slice-interleaved T_1_ mapping

^a^List of T_1_ mapping sequences is not exhaustive—more variants exist in the published literature that may not be recapitulated here

A T_1_ map is a two-dimensional (usually brightly colored) slice image where each image pixel displays the T_1_ relaxation time (ms) using a color look-up table to facilitate visual assessment [17]. Imaging at identical time points of the cardiac cycle is needed to yield co-registered images for curve-fitting and spatially resolved quantification of T_1_ [6]. Earlier T_1_ measurement approaches did not do this and became obsolete [18]. When combining raw images, some errors may therefore stem from RR-interval variability (arrhythmia, mistriggering), through-plane cardiac motion that is a normal part of longitudinal cardiac function, and diaphragmatic motion due to respiration. Automated non-rigid registration algorithms can correct for the position of source images to avoid some of this [19, 20]. Acquisition recommendations are now made to minimize other potential sources of errors in sequences and scan planning. For example, operators must aim to minimize partial volume effects by optimal slice orientation relative to the tissue, which is preferably orthogonal to the imaging plane to minimize obliquity. Proper adjustment of the shim volume and center frequency should be ensured to minimize off resonance artifacts [21]. A typical scan protocol is provided in the 2013 SCMR consensus statement [18].

T_1_ mapping is complex as different approaches are taken with different names. The original Look-Locker sequence developed in the 1970s [22] applied multiple inversion recovery pulses with different times-to-inversion, generating 20 distinct T_1_-weighted images. The inversion pulse inverted the net magnetization by 180° and was followed by multiple readout pulses interspersed with longitudinal magnetization recovery periods. However, as the relaxation curve was repeatedly perturbed by radiofrequency (RF) pulses of the imaging readout, an “apparent” T_1_ (T_1_*) was assessed and it required further correction for relaxation time measurement [23, 24]. The original Look-Locker was impractical for generating T_1_ maps, as acquisition, lasting 20 min, spanned multiple phases of the cardiac cycle [22]. Use of a new single-shot balanced steady-state free precession (SSFP) readout during diastole [25] permitted better signal-to-noise ratio and efficiency, intrinsic flow compensation [3], and consequently the development of the first *MO*dified *L*ook-*L*ocker *I*nversion Recovery (MOLLI) [6] approach in a single breath-hold of 17 heartbeats [26]. New MOLLI variants manipulate the prepulses and pauses between them. For example, the original MOLLIs used a 3(3*b*)3(3*b*)5 protocol, with numbers outside of parentheses indicating the number of images acquired after each magnetization preparation pulse, and numbers in parentheses indicating the length of the pause separating image acquisition and any subsequent magnetization preparation pulse, defined either in terms of number of recovery beats (*b*) or number of seconds (*s*). MOLLI’s bSSFP readout also estimates an “apparent” T_1_ (T_1_*) which is influenced by imaging RF pulses, so a Look-Locker correction is still needed to correct for it and deliver a more accurate T_1_ estimate [25]. Later versions, like the 5(3 *s*)3 variant [11], which shifts the bulk of image acquisition to the “beginning,” allow more time for recovery of longitudinal magnetization. Counting rest periods in seconds instead of recovery beats makes sequences more heart rate independent. Another approach, Shortened MOLLI [8] (ShMOLLI), uses a 5(1*b*)1(1*b*)1 scheme to acquire images over nine heartbeats making it more suitable for breathless patients [25]. The resultant dataset is however sparser and the one-beat pauses are insufficient to maintain compatibility with the theoretical model used in subsequent T_1_ estimation, for large T_1_ values [3]. ShMOLLI, therefore, employs a conditional fitting algorithm that includes the final two image acquisitions in the curve fitting routine only when the T_1_ estimate tends toward a smaller value [3]. The same Look-Locker correction as for MOLLI is applied. *SA*turation Recovery Single *SH*ot *A*cquisition [11] (SASHA) uses a saturation recovery instead of an inversion recovery preparation. Dephasing the whole imaging volume leads to depletion of the entire magnetization, alleviating the need for any rest periods. Because only one image is acquired after each magnetization preparation, the Look-Locker correction is not required and T_1_ can be estimated directly from pixel-wise curve fitting [3]. Unlike MOLLI, SASHA does not demonstrate heart rate dependence [26], but it can be less precise on account of the reduced dynamic range (90° vs. 180°). SASHA acquires 10 images in 10 heartbeats with the initial image lacking a saturation preparation [11]. *SA*turation *P*ulse *P*repared *H*eart-Rate Independent *I*nversion *RE*covery Sequence (SAPPHIRE) uses a hybrid combination of both inversion and saturation pulses that increases the dynamic range (a hybrid of MOLLI and SASHA, trying to get the best of both). Additional comparator sequences are elaborated in Table 1 **.**

## Biological basis of ECV

The myocardium can be considered as two main compartments: the “intracellular cellular volume” (ICV, 1 − ECV), dominated by myocytes but also including all other cells (fibroblasts, circulating red blood cells, etc.); and the “extracellular volume,” dominated water associated with the extracellular matrix but also including the intracapillary plasma volume [18]. The normal myocardial ECV value is around 25.3 ± 3.5% in health [27]. This is much higher than, for example, skeletal muscle, where the ECV may be 10%—myocardium has a lot more collagen (Tables 2 and 3). Various pathophysiological processes alter the ECV and ICV. We now know that athletic adaptation inducing left ventricular hypertrophy reduces the myocardial ECV, meaning that cellular hypertrophy is outweighing fibrosis increases [40]. The ECV may increase with fibrosis, edema, or other protein deposition (amyloid) [46]—or a combination [47]. However, increased capillary density or vasodilatation would also increase ECV, although to a smaller extent [48]. Therefore, ECV changes in isolation require interpretation.

**Table 2:** Typical ranges of native myocardial T_1_ in myocardial disease

Condition	Native T_1_^a^ [T; sequence; *n*]	*Z* value^b^	Reference
Aortic stenosis	1191 ± 34 [3 T; MOLLI; 20]	+0.4	Chin et al. 2014 [28]
Essential hypertension	955 ± 30 [1.5 T; ShMOLLI; 40]	–0.3	Treibel et al. 2015 [29]
Hypertrophic cardiomyopathy	1026 ± 64 [1.5 T; ShMOLLI; 46]	+1.7	Fontana et al. 2014 [30]
Dilated cardiomyopathy	1056 ± 62 [1.5 T; MOLLI; 29]	+0.9	aus dem Siepen et al. 2015 [31]
Acute myocardial infarction	1245 ± 75 [1.5 T; MOLLI; 40]	+9.8 ♦	Bulluck et al. 2016 [32]
Fabry disease	853 ± 50 [1.5 T; ShMOLLI; 38]	–3.6 ♦	Pica et al. 2014 [33]
Iron overload	863 ± 138 [1.5 T; ShMOLLI; 53]	–4.1 ♦	Sado et al. 2015 [34]
Light chain amyloidosis	1130 ± 68 [1.5 T; ShMOLLI; 79]	+4.8 ♦	Fontana et al. 2014 [30]
Transthyretin amyloidosis	1097 ± 43 [1.5 T; ShMOLLI; 85]	+3.8 ♦	Fontana et al. 2014 [30]
Acute myocarditis	1064 ± 37 [1.5 T; MOLLI, 61]	+6.2 ♦	Hinojar et al. 2015 [35]
Convalescent myocarditis	995 ± 19 [1.5 T; MOLLI; 67]	+2.8 ♦	Hinojar et al. 2015 [35]

T_1_ values per disease were derived from at least one representative work in the published literature (other relevant works exist that have not been referenced here). Reported ranges are only applicable to the sequence, imaging protocol, field strength, and scanner configuration used by the group and are not necessarily immediately generalizable across centers [18]. The native T_1_ signal in some diseases (annotated by “♦”) shows a large deviation (multiple SDs) from normality, so T_1_ mapping is bound to be more robust here as the pathology-related T_1_ change trumps any “normal” biases that confound T_1_ estimates. In other heart muscle diseases, however (e.g., hypertensive heart disease, aortic stenosis), where T_1_ changes are less dramatic, biases in T_1_ estimates may become major signal pollutants, so pathology-related T_1_ differences may not be realistically resolvable except through large, standardized studies

*SD* standard deviation, *T* Tesla. Other abbreviations as in Table 1

^a^Reported in milliseconds as mean ± SD. Defines field-strength (T), sequence used, and sample size (*n*) of the diseased cohort

^b^Number of SDs by which a particular disease’s mean T_1_ value lies above or below the healthy control mean T_1_ reported by the group in the same study

**Table 3:** Measured ECV relationship in some heart muscle disease

Condition	ECV^a^ (%) [T; *n*]	Reference
Acute myocardial infarction	⇑ 56 ± 1.4 [1.5 T; 39]	Kidambi et al. 2016 [36]
Aortic stenosis^||^	↔ 24.3 ± 1.9 [3 T; 50] ⇑ 28.3 ± 1.7 [3 T; 20]	Singh et al. 2015 [37]
		Chin et al. 2014 [28]
Hypertrophic cardiomyopathy	⇑ 37.1 ± 10.1 [3 T; 50]	Swoboda et al. 2017 [38]
Dilated cardiomyopathy	⇑ 27 ± 4 [1.5 T; 29]	aus dem Siepen et al. 2015 [31]
Systolic heart failure	⇑ 31.2, 29.0–34.1^~^ [3 T; 40]	Su et al. 2014 [39]
Heart failure preserved ejection fraction	⇑ 28.9, 27.8–31.3^~^ [3 T; 62]	Su et al. 2014 [39]
Athletic adaptation	↓ 22.5 ± 2.6 [1.5 T; 30]	McDiarmid et al. 2016 [40]
Fabry disease	↔ 21.7 ± 2.4 [1.5 T; 31]	Thompson et al. 2013 [41]
Iron overload	⇑ 31.3 ± 2.8 [1.5 T; 19]	Hanneman et al. 2016 [42]
Light chain amyloidosis	⇑ 54 ± 7 [1.5 T; 92]	Fontana et al. 2015 [43]
Transthyretin amyloidosis	⇑ 60 ± 7 [1.5 T; 44]	Fontana et al. 2015 [44]
Acute myocarditis	⇑ 30, 27–32^§^ [1.5 T; 48]	Bohnen et al. 2017 [45]

*ECV* extracellular volume. Other abbreviations as in Table 2

↑ increase, ↓ decrease, ⇑ marked increase, ↔ static

^a^Cited ECV values (%) are as mean ± SD except where otherwise stated. Field-strength (T) and sample size (*n*) are additionally provided. ECV ranges per disease were derived from at least one representative work in the published literature (other relevant works exist that have not been referenced here)

^||^Conflicting data currently

§Median, first, and third quartiles

~Mean, interquartile range

Mathematical derivation of the ECV (Eq. 1) relies on (1) a number of assumptions (including the fast-exchange limit as reviewed elsewhere) [3], (2) measurement of the partition coefficient (the bold right half of Eq. 1, also known as ***λ***), and (3) the patient’s hematocrit (*Hct*) representing the cellular fraction of blood [2].

1 $$ ECV=\left(1- Hct\right) \times \left.\left(\frac{\frac{1}{\boldsymbol{T}1\ \boldsymbol{myocardium}\ \boldsymbol{post}-\boldsymbol{GBCA}}-\frac{1}{\boldsymbol{T}1\ \boldsymbol{myocardium}\ \boldsymbol{native}}}{\frac{1}{\boldsymbol{T}1\ \boldsymbol{blood}\ \boldsymbol{post}-\boldsymbol{GBCA}}-\frac{1}{\boldsymbol{T}1\ \boldsymbol{blood}\ \boldsymbol{native}}}\right)\right\}\ \lambda $$

## T_1_ mapping and ECV in selected high signal diseases

### Lipid storage disease

**Fig. 3:**
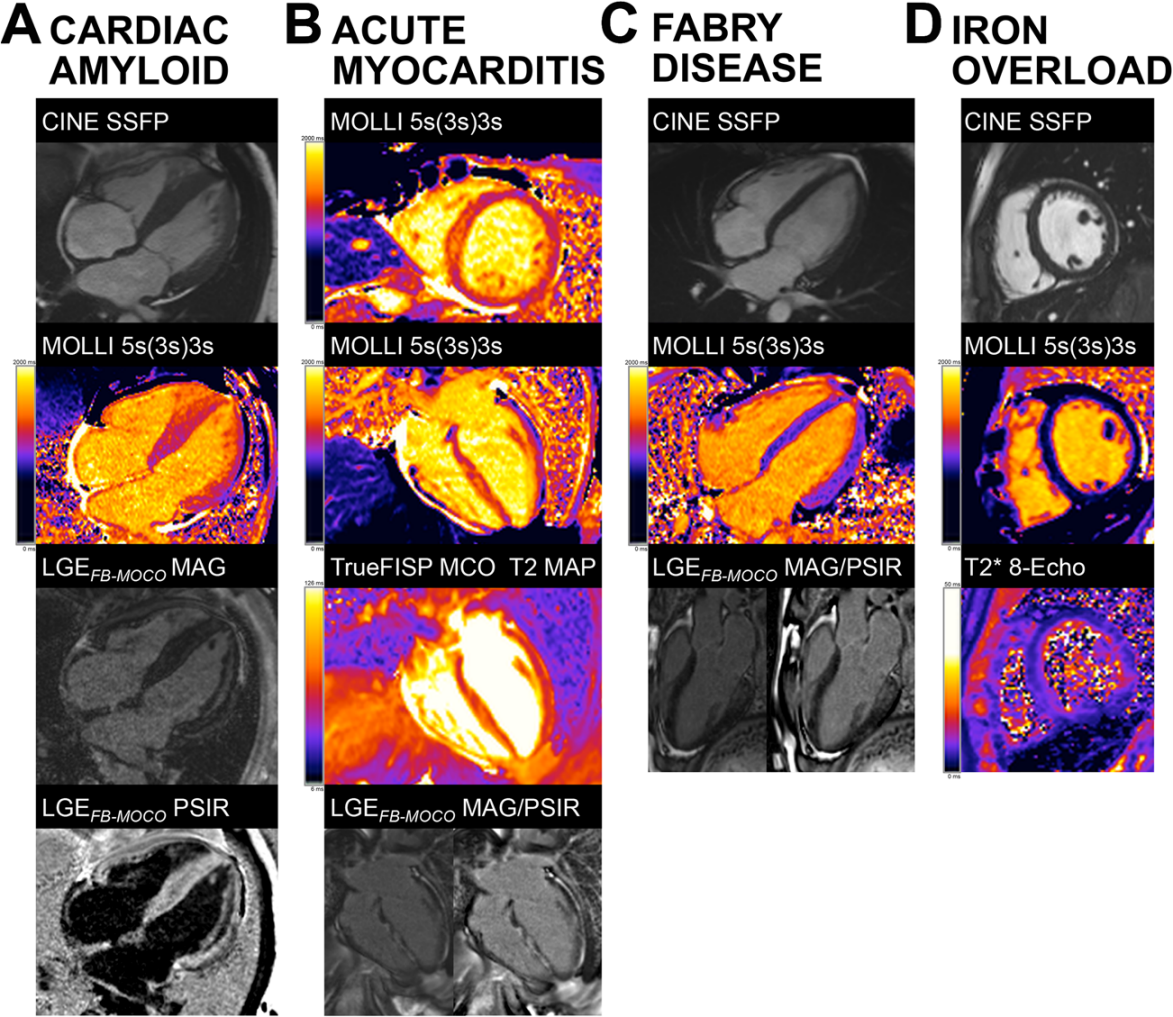
The practical clinical utility of T_1_ mapping in selected heart muscle diseases. **a** Cardiac amyloidosis showing marked septal thickening. There is high native T_1_ (1270 ms in the septum) and near transmural and myocardial enhancement and severe expansion of the ECV is predicted (in-line synthetic ECV 49). **b** Acute myocarditis showing abnormal myocardium tissue characterization with high native T_1_ (1345 ms in the septum) and T_2_ (71 ms in the septum), extensive LGE, and high ECV (in-line synthetic ECV 54). **c** Fabry disease showing no LV hypertrophy (early-phenotype) and low native T_1_ globally (877 ms) except for the basal infero-lateral wall, co-locating with no-ischemic fibrosis. ECV is normal. **d** Cardiac iron overload in a thalassemic patient showing T_2_* 8 ms and native T_1_ reduction to 670–750 ms by MOLLI. *ECV* extracellular volume fraction, *FB* free-breathing, *FISP* fast imaging with steady-state precession, *GBCA* gadolinium-based contrast agent, *LGE* late gadolinium enhancement, *LV* left ventricle, *MOCO* motion-corrected, *MOLLI* modified Look-Locker inversion recovery, *PSIR* phase-sensitive inversion recovery, *SSFP* steady-state free precession

Fabry disease (FD) is an intracellular lysosomal storage disease caused by the accumulation of globotriaosylceramide in tissues due to a deficiency in the enzyme α-galactosidase A [49]. Cardiac involvement causes concentric LVH, arrhythmias, and heart failure, and it is the major cause of mortality [50]. This lipid (in classic lamellar bodies) probably causes the native myocardial T_1_ to be low, and the result is that T_1_ mapping can reliably differentiate between FD, other forms of LVH, and healthy controls [51]. T_1_ lowering is seen in 50–60% of subjects before LVH (Fig. 3c), so it is a biomarker of early cardiac involvement [51], correlating with reduced global longitudinal strain by echocardiography [33]. Because ECV primarily reflects extracellular interstitial disease, it misses the intracellular lysosomal storage, but there may be future roles for late phenotype development as diffuse fibrosis starts [41]. In the infero-lateral wall, where FD has LGE, segmental T_1_ and T_2_ elevation may occur (where the pseudo-normalized or elevated T_1_ is likely due to the effects of replacement fibrosis dominating the fatty-related T_1_ decrease) and these correlate with blood troponin suggesting that chronic inflammation may be contributing [52]. Enzyme replacement therapy (ERT) for FD may be most beneficial if started sufficiently early, before the establishment of permanent changes [53], but ERT is expensive and early initiation carries societal implications. T_1_ mapping, capable of detecting early cardiac involvement in FD, could therefore have a major role in guiding timing of commencement of ERT and drug monitoring [33].

### Myocarditis

Myocardial inflammation is a key step in the development of multiple cardiac diseases. CMR tissue characterization has major potential in its diagnosis. The 2009 “Lake Louise” myocarditis criteria, drafted before mapping was widespread, require the presence of two out of the following three findings: increased myocardial edema by T_2_-weighted imaging, non-ischemic mid-wall LGE, and hyperemia/capillary leak on early gadolinium enhancement imaging [54]. These are known to be insensitive [55]. Mapping helps. Combining ECV (ECV cut-off ≥27%) with LGE data significantly improves the diagnostic accuracy (90% compared with 79% [54]), and normal ECV has been shown to rule out myocardial damage with a high degree of certainty [56]. Native T_1_ detects both intracellular and diffuse myocardial change (Fig. 3b), so it has a role in grading the severity and stage of myocardial inflammation [35, 57]. The MyoRacer trial suggests that the most useful imaging tools for confirming or refuting a diagnosis of acute myocarditis are native T_1_ mapping, followed by T_2_ mapping, ECV, and Lake Louise criteria in this descending order. By contrast, only T_2_ mapping showed diagnostic utility in chronic myocarditis [58]. A multiparametric CMR approach toward myocarditis is envisaged: one which exploits T_1_ mapping and ECV as well as T_2_ mapping, T_2_-weighted imaging, early gadolinium enhancement, LGE, and Lake Louise criteria to quantifying the extent of inflammation and distinguish between acute and chronic myocardial injury [59].

### Myocardial infarction

Acute and chronic infarct imaging is done by standard LGE techniques, but T_1_ mapping and ECV provide complementary information, both diagnostically and prognostically. In acute myocardial infarction (MI), myocardial edema elevates the native T_1_ signal and the ECV. Native T_1_ in the infarct core can predict 6-month post-ST-elevation myocardial infarction (STEMI) mortality even after adjustment for LV ejection fraction [60], and in the remote myocardium, native T_1_ is independently associated with LV systolic dysfunction [61]. In reperfused acute MI, acute infarct ECV, unlike standard LGE, is independently associated with ejection fraction and convalescent infarct global strain, suggesting it is a better predictor of LV functional recovery [36, 62]. Native T_1_ may also identify the area at risk and salvaged myocardium [63] better than T_2_-weighted imaging can. In chronic MI, native T_1_ and ECV are increased, but values are lower than those observed in acute MI [64]. Native T_1_ values in chronic MI by widely used bSSFP mapping methods should be interpreted with caution as T_1_ values may be subject to additive or subtractive bias when water and fat coexist in the myocardium—intramyocardial fat due to lipomatous metaplasia in chronic myocardial scar potentially predisposes to such T_1_ biases [66]. In the field of stress perfusion CMR for ischemia, T_1_ mapping of the spleen is being explored as a surrogate indicator of adequacy of vasodilator stress with adenosine [67]. The splenic blood flow paradoxically reduces during the course of adenosine myocardial vasodilatation and native splenic T_1_ decreases as a result. This makes native splenic T_1_ in the course of the adenosine infusion (and before GBCA administration) a potential surrogate marker of stress adequacy [67].

### Cardiac amyloidosis

The ventricular myocardium is affected by immunoglobulin light chain (AL) and transthyretin (ATTR) amyloidosis, which has two subtypes, wildtype and mutant [68]. Amyloid deposits and infiltrates the myocardial interstitium and is the major determinant of outcome [43]. Amyloidosis on LGE has characteristic appearances, particularly with the phase-sensitive inversion recovery technique. In early disease, the LGE may be normal. Later, global subendocardial LGE (but more prevalent at the base) may occur, associated with blood and myocardium nulling together. Later still, transmural LGE appears [69]. However, native myocardial T_1_ and ECV may have more discriminatory and predictive power than LGE [46, 70], and they change before LGE [71]. The current working hypothesis is that the ECV can be higher in ATTR due to higher cell volume (derived as 1 − ECV × myocardial mass), indicating concomitant myocyte hypertrophy [44]. Conversely, native T_1_ (Fig. 3a) can be higher in AL due to the influence of myocardial inflammation [30]. As treatment options differ between AL and ATTR, differentiating between the two by T_1_ mapping and ECV is clinically important [72].

### Iron overload

Iron shortens all three CMR relaxation times—T_1_, T_2_, and T_2_* [73] (Fig. 3d). T_2_* at 1.5 Tesla (T) (but not at 3 T [74]) is the gold standard for myocardial iron overload assessment and has transformed clinical outcomes when it is used as it can target chelation therapy to where it is needed most [75]. T_1_ mapping has potential here as well and can serve as a complementary tool [76]. Native myocardial T_1_ correlates well with T_2_* but has the added advantage of greater reproducibility and sensitivity, and it can detect lower myocardial iron levels potentially missed by T_2_* [34, 42, 77–79]. In thalassemia major, for example, native T_1_ detected cardiac iron overload in a third of cases missed by T_2_* [76].

Challenges facing the roll-out of native myocardial T_1_ for cardiac iron assessment include the known variation of absolute T_1_ between sequences and scanners [78] and its non-specificity—its susceptibility to alter in a large number of heart muscle diseases. In this respect, T_2_* is more disease specific [80]. This advantage should not be overstated—the T_1_ changes of significant iron completely swamp all other pathologies—the T_1_ can lower by an impressive 25 standard deviations in severe iron overload, for example. The ECV can be used in iron overload, although there are concerns when iron loading is significant. The ECV can be increased in thalassemia major patients with documented cardiac iron overload, and it correlates with T_2_* but not with LV systolic function and global longitudinal strain [42]. The impression is that cardiac iron could be transitioning to a fibrotic phenotype, although there is little autopsy evidence for this [81].

## T_1_ mapping and ECV in selected modest signal diseases

### Dilated cardiomyopathy

In dilated cardiomyopathy (DCM), diffuse myocardial fibrosis may be a prominent feature during disease progression and cardiac remodeling, which eludes depiction by LGE imaging. Early myocardial fibrosis detected by native T_1_ mapping in DCM [31] can predict adverse outcomes [82] allowing for risk stratification and for the initiation of timely and appropriate management. However, the T_1_ signal change in DCM is not large and conventional T_1_ mapping approaches have in-plane resolution limitations when applied to thin-walled hearts (a prevalent phenotype in DCM [83]). Native T_1_ is prolonged in DCM and inversely correlated with wall thickness [84, 85] where confounding by partial volume effects may play a part. During the early (subclinical) stages, hearts may have normal LV wall thickness values (~10 mm), so a conventional T_1_ mapping sequence could potentially be used, but once the DCM phenotype manifests (often with an increase in overall LV mass), wall thickness may or may not decline with significant partial volume implications. ECV was shown to correlate with clinical prognosis in DCM [86] and with LV systolic dysfunction [87], and although it is recommended in the 2013 T_1_ mapping consensus document [18], it is still not accurate enough to be of proven utility for early diagnosis and risk stratification in DCM [18, 31, 88]. T_2_ mapping can detect myocardial inflammation that appears to play an important role in non-ischemic DCM [89].

### Hypertrophic cardiomyopathy

Myocardial disarray, small vessel disease, and fibrosis are histopathological hallmarks of familial sarcomeric HCM. In HCM, LGE is a risk factor for heart failure and an additional risk factor for SCD [90]. T_1_ mapping can have additive value [91]. Native T_1_ is modestly elevated in HCM as compared to healthy controls and highest in the areas of maximal hypertrophy [90]. T_1_ may also be elevated in HCM patients without overt LV hypertrophy, suggesting potential clinical utility as an early disease biomarker [84]. Native T_1_ was better than ECV at discriminating HCM from hypertensive heart disease [92] and it identified subclinical HCM in sarcomere gene mutation carriers [92], although some of these have rather thin walls and crypts that could lead to partial volume effects and native T_1_ correlated with LV remodeling and global systolic function [85]. ECV cannot discriminate between overt HCM and DCM being similarly elevated in both, suggesting a final common pathway of interstitial change [93], but it can differentiate between sarcomeric HCM and athletic heart as the latter exhibits reduced ECV in the hypertrophied segments [94].

### Valvular heart disease

Most T_1_ mapping studies for valvular heart disease have focused on aortic stenosis (AS). AS is associated with two forms of myocardial fibrosis: diffuse (interstitial) fibrosis that may appear prior to symptom manifestation and architectural change, and the more focal, late irreversible replacement fibrosis. Our understanding of fibrosis in AS is incomplete. Some fibrosis is clearly advantageous, but maladaptive fibrosis also occurs and the myocardial adaptation to the narrowed valve is key to the clinical impact [95]. T_1_ mapping can quantify the diffuse myocardial fibrosis in AS providing an indication of AS severity and cardiac function [37, 96] and histopathological correlation has been achieved [74, 97]. Mild to moderate diffuse fibrosis in AS has been linked to postoperative LVH reduction and better symptomatic improvement compared to severe fibrosis at baseline [98]. Native T_1_ and ECV were shown to be increased in AS [28] especially in patients with more abnormal patterns of LV remodeling, and they tracked the prognostic biomarker n-terminal pro-brain natriuretic peptide [29], but in another study of asymptomatic moderate/severe AS patients, native T_1_ and ECV did not differ significantly from those in age-matched controls at 3 T [37].

Current guidelines classify AS severity mostly by echocardiography via trans-valvular pressure and aortic valve area measurements, and intervention is recommended based on LV ejection fraction and the presence of symptoms, ignoring the myocardial state, in spite of myocardial fibrosis having been shown to determine outcomes in AS patients [99] and ECV demonstrating prognostic value post-tissue aortic valve replacement [100]. The prognostic value of ECV in AS has recently been demonstrated [101] and the “iECV,” derived from the product of ECV and body surface area-indexed LV end-diastolic volume, showed good correlation with histology [101]. Diffuse fibrosis assessment by T_1_ mapping in chronic mitral regurgitation may also have clinical utility to guide timing of intervention [102].

## Biomarker roadmap for T_1_ mapping

**Fig. 4:**
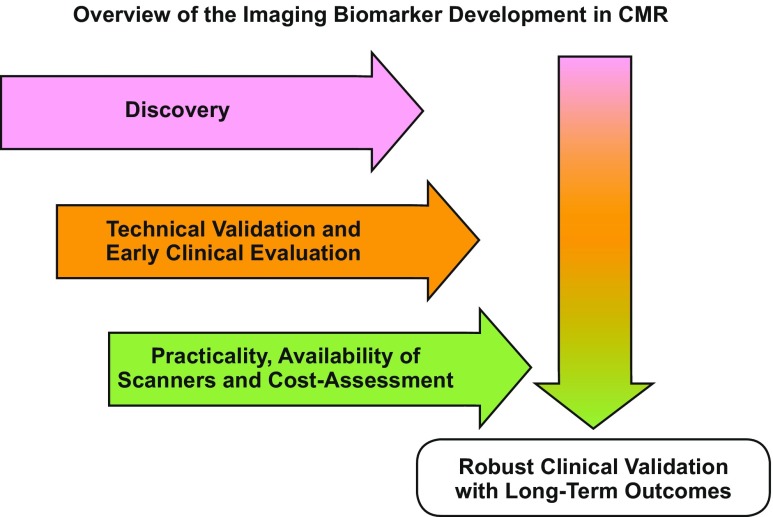
Overview of imaging biomarker roadmap for T_1_ mapping. The technical and early clinical validation of imaging biomarkers often occur in tandem. Cost-effectiveness and usability must be assessed for the biomarker to have the potential of full translational application. In parallel, prognostic assessment with hard outcomes must occur before routine integration into patient care

Familiar imaging biomarkers used daily in cardiac imaging include LV ejection fraction, wall thickness, and left atrial size. New imaging biomarkers such as T_1_ mapping and ECV are typically first established as useful complementary tools for new biological insights before becoming surrogate secondary endpoints in clinical studies. They must then cross the “translational gap” before they can become clinical decision-making tools [103] (Fig. 4). Therefore, for T_1_ mapping and ECV, three parallel, not entirely sequential processes, are needed: technical validation (e.g., through the use of phantoms [103, 104]), biological/clinical validation, and cost-effectiveness analysis. We are still missing cost-effectiveness studies for T_1_ mapping and ECV—not every T_1_ mapping sequence will have commercial viability as a diagnostic product in healthcare systems, although some sequences certainly will. T_1_ mapping cost-effectiveness studies are needed to inform on this dichotomy. The funded research agendas of individual centers make it easier to carry on with adding layers of T_1_ mapping innovation rather than halt the advancement, and scrutinize old work for cost-effectiveness, that may well end up generating unwelcome results. Even those T_1_ mapping sequences found to lack commercial viability as products may still have niche roles in the research setting, justifying the development of new models to oversee their continued research and development funding, and regulation. Large-scale health-economic considerations and cost-effectiveness studies in T_1_ mapping, when they happen, will also need to consider the broader portfolio of competitor tests that include other CMR (e.g., LGE, T_2_ mapping) and non-CMR imaging biomarkers, as well as biospecimen-derived biomarkers (e.g., troponin, N-terminal pro-brain natriuretic peptide, etc.) [105].

Furthermore, we need standardization and centrally coordinated accreditation systems for T_1_ mapping sites [105]. The issues of standardization and inter-operability is important for T_1_ mapping as measurements differ between CMR scanners, manufacturers, field strengths, protocols, pulse sequences [106], patient characteristics [107], and other factors. Depending on the sequence used, T_1_ mapping has specific limitations (see Table 1) that innovative approaches keep trying to address with encouraging results. Partial-volume effects at the interface between myocardium and blood-pool result in reduced accuracy and reproducibility [83, 108] and dark-blood preparation as well as systolic T_1_ mapping have been proposed as potential solutions to overcome these issues [108, 109]. Elaborate post-processing using improved modeling of the perturbed inversion curve has been studied to increase the accuracy of inversion-recovery-based T_1_ times [9, 110]. Saturation recovery methods were shown to improve the accuracy of T_1_ measurements compared to MOLLI, albeit at the expense of precision. Reconstructions with a reduced number of fit parameters have been proposed to trade off some of the precision loss against a slight drop in accuracy [83, 111]. Alternatively, SAPPHIRE can be employed, which through the use of a combined inversion/saturation recovery approach allows accurate T_1_ estimation without sacrificing as much of the precision as SASHA [107, 112]. Other efforts have addressed the RR-interval sensitivity of T_1_ mapping to improve its performance in the presence of arrhythmias such as atrial fibrillation [113]. Free-breathing T_1_ mapping sequences are being proposed to overcome motion artifact in sicker patients unable to breath-hold [111] coupled with advances in motion-correction algorithms [20, 114]. Lastly, to increase imaging efficiency and improve specificity beyond conventional T_1_ mapping, several methods for joint estimation of parameters have recently been explored [115, 116].

## Conclusion

T_1_ mapping and ECV of the heart are transforming contemporary CMR through their research and potential clinical applications. These biomarkers have potential to accurately inform clinical decision-making, but like all other biomarkers, they must first survive rigorous scrutiny, validation, and qualification. In spite of the research outputs and excitement within the CMR community, to date, although there has been a first consensus statement [18] with a second one pending, T_1_ mapping has yet to enter disease-specific guidelines (this may be pending for myocarditis). Still clinical utilization is proceeding with the use of these tools daily in many centers, so more is needed including a wider range of research (technical, translational, standardization) and further consensus/summary processes [117] to illuminate the T_1_ mapping field. Roadmapping these excellent biomarkers into healthcare for evidence-based patient management is an arduous, time-consuming, but important task. The CMR community needs such guidance.
